# Targeting immune cells for cancer therapy

**DOI:** 10.1016/j.redox.2019.101174

**Published:** 2019-03-20

**Authors:** Sin Yee Gun, Sharon Wei Ling Lee, Je Lin Sieow, Siew Cheng Wong

**Affiliations:** a Singapore Immunology Network, A*STAR, Singapore; b BioSystems and Micromechanics IRG, Singapore-MIT Alliance for Research and Technology, Singapore; c Department of Microbiology and Immunology, Yong Loo Ling School of Medicine, National University of Singapore, Singapore; d School of Biological Sciences, Nanyang Technological University, Singapore

**Keywords:** Cancer, Immunotherapy, Nanoparticles, Immune checkpoint, Inflammation

## Abstract

Recent years have seen a renaissance in the research linking inflammation and cancer with immune cells playing a central role in smouldering inflammation in the tumor microenvironment. Diverse immune cell types infiltrate the tumor microenvironment, and the dynamic tumor-immune cell interplay gives rise to a rich milieu of cytokines and growth factors. Fundamentally, this intricate cross-talk creates the conducive condition for tumor cell proliferation, survival and metastasis. Interestingly, the prominent impact of immune cells is expounded in their contrary pro-tumoral role, as well as their potential anti-cancer cellular weaponry. The latter is known as immunotherapy, a concept born out of evidence that tumors are susceptible to immune defence and that by manipulating the immune system, tumor growth can be successfully restrained. Naturally, a deeper understanding of the multifaceted roles of various immune cell types thus contributes toward developing innovative anti-cancer strategies. Therefore, in this review we first outline the roles played by the major immune cell types, such as macrophages, neutrophils, natural killer cells, T cells and B cells. We then explain the recently-explored strategies of immunomodulation and discuss some important approaches via an immunology perspective.

## 1 Introduction

In the year 2000, Hanahan and Weinberg defined six traits that are shared by all cancers, showcasing that the complexity of cancer can be rationalized into just a few organizing principles [1]. These hallmarks included genome instability, self-sufficient growth and apoptotic evasion [2]. A role for inflammation in cancer development was proposed in 1893, when Rudolf Virchow observed the presence of immune cells in neoplastic tissues [3]. It was not until 2011, however, that a role for the immune system in cancer development was formally acknowledged, with the inclusion of immune system evasion and inflammation as additional hallmarks. Inflammation orchestrates the host defence to pathogens and tissue injury by mediating tissue repair and regeneration. Although this defence mechanism is essential to protect the host from infection and injury, inflammation may also serve as a double-edged sword, whereby an over-activated innate immune response can lead to autoimmune diseases such as systemic lupus erythematosus [4]. We now know that inflammation can also trigger tumor initiation, enhance tumor progression and facilitate cancer-cell dissemination [[5], [6], [7], [8]].

The past decade has seen a renaissance in the research linking inflammation and cancer [9]. Cancer and inflammation are linked by two pathways: the extrinsic and intrinsic pathways [10]. In the extrinsic pathway, tumor initiation and development are triggered by inflammation or infection. In the intrinsic pathway, somatic alterations and genetic mutations activate signaling pathways that lead to an inflammatory response [10]. Both pathways can converge and regardless of the origin, mediators and effectors of inflammation (such as inflammatory cells), cytokines and growth factors create an optimal environment for tumor-cell proliferation, survival and metastasis [3,11]. The indispensable role of immune cells in supporting tumor proliferation, survival and metastasis is now being uncovered [12].

Cancer cells produce cytokines and chemokines that attract a diverse immune-cell infiltrate composed of mostly but not exclusively macrophages, neutrophils and lymphocytes [12]. These infiltrating immune cells can produce cytotoxic mediators, such as reactive oxygen species (ROS), matrix metalloproteinases and cytokines (tumor necrosis factor-α (TNF-α), interleukins and interferons) [12]. Persistent activation of the immune system and failure of the inflammatory response to resolve, however, results in chronic inflammation. The chronic inflammatory microenvironment fosters genomic lesions and promotes tumor growth. One effector mechanism includes the production of free radicals by the host — such as reactive oxygen intermediates (ROI), hydroxyl radicals, superoxide, reactive nitrogen intermediates (RNI), nitric oxide and peroxynitrite [13]. Notably, ROI and RNI increase the risk of DNA mutations via oxidative stress and nitration of DNA bases [13]. Finally, failure of cell death and repair programs in chronically inflamed tissues leads to continuous DNA replication and cellular proliferation.

Cancer immunotherapy was conceived in the late nineteenth century when Coley injected bacterial products (“Coleys toxin”) into an inoperable sarcoma and observed tumor shrinkage [14]. This was the first evidence that tumors are susceptible to the host immune response and manipulation of the immune defence can successfully restrain tumor growth. Modern immunotherapy strategies have been developed based on various approaches, including boosting the anti-tumoral response or relieving immunosuppression. In this review we first outline the role of the major immune cells involved in cancer progression, including macrophages, neutrophils, natural killer (NK) cells, T cells and B cells. We then explain the latest immunological strategies developed thus far to manipulate anti-tumor response and areas in which cancers may be targeted from an immunology perspective.

## 2 Immune cells in cancer

### 2.1 Macrophages

Macrophages are a prominent immune-cell population involved in diverse aspects of immunity and immune homeostasis. While these essential immune cells help mediate normal physiological processes, such as wound healing, response to infection and normal tissue homeostasis, they can also promote disease conditions such as autoimmune disorders, atherosclerosis and tumorigenesis [15].

High levels of cellular plasticity and diversity allow macrophages to change phenotype and polarize into different subsets in response to a wide variety of environmental cues [15]. According to the binary polarization principle, there are two macrophage polarization states: M1 and M2. M1 “classically activated” macrophages are activated by interferon gamma (IFN-γ) and lipopolysaccharide. These macrophages produce pro-inflammatory cytokines, nitric oxide and/or ROI to mount an immune response against bacteria and viruses [15]. Consequently, these cells provide a favourable response against disease pathogens. M2 “alternatively activated” macrophages are activated by cytokines, including interleukin (IL)-4 and IL-10. These macrophages produce anti-inflammatory cytokines and are involved in wound healing and tissue repair [15]. However, these cells also induce suppressive immunity against parasites and tumor cells, promote angiogenesis and matrix remodelling that leads to tumor progression and metastasis [16]. Consequently, the presence of these cells are unfavourable to patients with cancer.

Recent data suggest that this binary macrophage classification system is insufficient to account for the diverse cellular subtypes, phenotypic changes and effector functions observed *in vivo* [17]. Indeed, we and others have shown that *in vitro* tumor-conditioned tumor-associated macrophages (TAMs) exhibit a mixed M1/M2 macrophage phenotype, expressing both M2 (CD163 and CD206) and M1 (IL-1β, IL-6, TNF-α, and CCL3) markers [18,19].

*Tumor associated macrophages* TAMs promote cancer metastasis through a number of mechanisms including promoting angiogenesis, inducing tumor growth and enhancing tumor-cell migration and invasion [20]. Thus unsurprisingly, clinical data have shown a correlation between the number of TAMs in the tumor microenvironment (TME) and poor prognosis for breast, prostate, ovarian, cervical, endometrial, esophageal and bladder cancers [20]. TAMs express vascular endothelial growth factor-C (VEGF-C), VEGF-D and VEGFR-3, all of which are essential for lymphatic vessel formation, angiogenesis and metastasis [21]. Indeed, TAM depletion using clodronate liposomes and angiogenesis inhibition using anti-VEGF antibodies significantly reduces tumorigenesis [22]. TAM depletion in the TME may, therefore, be a potential anti-tumoral strategy to inhibit tumor progression.

TAMs also promote tumorigenesis through immunosuppression and inhibiting anti-tumoral immunity as shown both *in vitro* and in mouse studies. TAMs can enhance tumor evasion of the immune surveillance system in two ways: (1) by directly inhibiting anti-tumoral cytotoxic CD8^+^ T cell responses via PD-L1/PD-L2 expression [23]; and (2) by secreting immunosuppressive cytokines and proteases such as arginase-1, IL-10, TGF-β and prostaglandins, which prevent T cell activation [17,24,25].

### 2.2 Neutrophils

Many *in vitro* and *in vivo* studies have highlighted that neutrophils, like macrophages, also have critical roles in mediating tumor progression [26]. Polymorphonuclear neutrophils are the most abundant circulating leukocyte in humans. They are innate immune cells involved in the first line of defence against infections, and thus have an indispensable role in the inflammatory response. During an infection, activated neutrophils release proteinases into the microenvironment that damage surrounding tissues. They also produce cytokines and chemokines that recruit other inflammatory cells and alter the immune response [27].

However in cancer settings, these cells are not mere bystanders; neutrophil recruitment and activation has been observed in tumors and reflects a state of host inflammation [2]. Neutrophils are involved in various stages of tumorigenesis including tumor initiation, proliferation and metastasis [28,29]. They infiltrate tumors in large numbers and both *in vitro* studies as well as patient studies that were performed in the 1980s showed that neutrophils can kill tumor cells and mediate tumor cytotoxicity [30,31]. The pro-tumoral functions of neutrophils, however, have only been shown relatively recently. As such, the current literature describes tumor-associated neutrophils (TANs) as a double-edged sword, performing both anti-tumoral and pro-tumoral functions [26,[32], [33], [34]]. Tumor growth initiation can be induced by ROS, reactive nitrogen species or protease release by TANs [35]. ROS production by neutrophils is an effective mechanism to kill microorganisms and is important in the early stages of tumor development, where ROS-induced apoptotic signaling kills tumor cells [36]. However, in cases where neutrophil-derived ROS is not sufficient to kill tumor cells, it can indirectly promote tumor growth through DNA damage and genotoxicity [37].

**Fig. 1:**
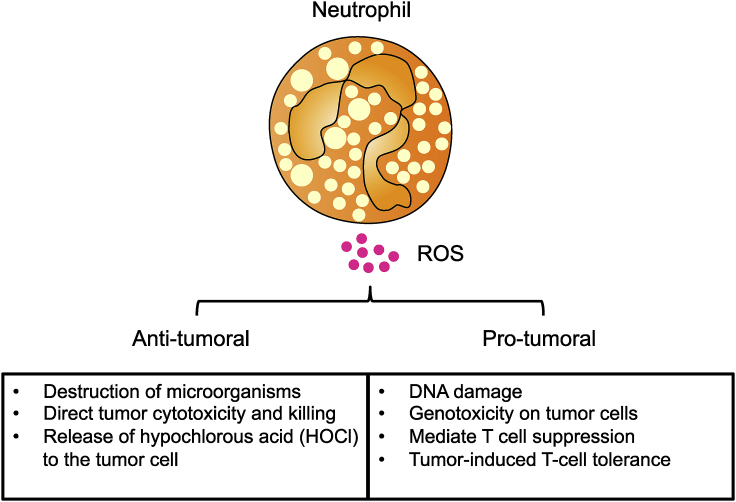
**The positive and negative effects of reactive oxygen species (ROS) on tumor growth.** On the anti-tumorigenic side of the balance: ROS induce the killing of microorganisms, apoptosis of tumor cells and the release of hypochlorous acid (HOCL) which directly promote tumor cytotoxicity. On the other pro-tumorigenic side of the balance: ROS induces DNA damage, genotoxicity, mediate T cell suppression as well as T cell tolerance, leading to the initiation of tumor growth. The delicate balance between ROS production and oxidative stress can modulate the pro- or anti-tumorigenic tumor microenvironment.

The impaired immune response reported in cancer patients correlates with exposure to oxidative stress. As such, the elevated ROS levels produced by activated neutrophils are considered an obstacle for effective cancer immunotherapy [38]. In advanced cancer patients, activated TANs and their production of hydrogen peroxide is the underlying cause of impaired T cell function and suppression [39]. Hydrogen peroxide suppresses cytokine production by normal T cells and reduces T cell receptor zeta chain expression, leading to immunosuppression [39]. For example, the *in vitro* exposure of memory and effector CD45RO^+^ T cells to ROS blocks their NF-κB activation and reduces Th1 cytokine production [38]. Furthermore, murine studies have demonstrated that ROS can lead to CD8^+^ T cell tolerance by nitration of tyrosines within the TCR/CD8 complex and subsequently preventing specific peptide–MHC dimers from binding to CD8^+^ T cells [40]. Consequently, CD8^+^ T cells are unable to bind the pMHC and respond to the specific peptide, resulting in tumor-induced T cell tolerance and tumor escape. In a study using patient samples, Arginase 1, a known immuno-suppressor of the immune system, inhibits T cell proliferation and activation by rendering T cells unresponsive to CD3/TCR stimulation [41]. TAN-derived arginase 1 thus also promotes T cell suppression [41]. Taken together these mechanisms explain how oxidative stress, ROS and arginase 1 can mediate anti-tumoral T cell suppression in the TME and how they may be modulated for effective immunotherapy (Fig. 1).

Neutrophil migration is predominantly mediated by the chemokine receptor CXCR2. Murine studies have shown that TANs are attracted by CXCR2 ligands, such as CXCL1, CXCL2 and CXCL5 produced in the TME [42]. Indeed, CXCR2 deficiency halts neutrophil recruitment and CXCR2 inhibition reduces colitis-associated tumorigenesis [43]. CXCR2 may, therefore, be a pro-tumorigenic chemokine receptor that aids pro-inflammatory leukocyte recruitment into the inflammatory TME. Inhibiting CXCR2 may have therapeutic effects in cancer [43].

Similar to macrophages, tumor-associated neutrophils (TANs) exhibit skewed phenotypes that can be classified as N1-like or N2-like neutrophils. Early infiltrating murine TANs are N1-like neutrophils as they exhibit a pro-inflammatory and anti-tumoral phenotype [26]. However, as the tumor progresses, cytokines, predominantly transforming growth factor beta (TGF-β) and the inflammatory TME skew TANs to become N2-like neutrophils where they exhibit a pro-tumoral phenotype. These N2-like neutrophils favour tumor progression and metastasis through the release of VEGF to promote angiogenesis, and the expression of arginase 1 to suppress cytotoxic T cell anti-tumoral activity [44]. A study conducted by Sagiv and colleagues, found that murine neutrophils could also be distinguished according to their density [32]. High-density neutrophils represent N1-like cells and low-density neutrophils (LDNs) represent N2-like pro-tumoral cells; LDNs can be further subdivided into mature and immature cells [32]. Taken together, these studies illustrate the extent of neutrophil plasticity and the phenotypic changes that occur according to environmental stimuli. Hence the modulation of neutrophils to a more anti-tumoral phenotype would be a potential therapeutic avenue.

### 2.3 NK cells

NK cells are innate cells with cytotoxic capacity. Clinical correlation studies revealed that tumors with low NK activity are associated with poor survival outcomes [45] and tumor control [46] in laryngeal and gastric carcinoma, respectively. NK lytic potential is mediated either through lytic granule release or death signal expression. To perform this function, NK cells probe other cells with activating (which identify stress-induced or foreign ligands) and inhibitory (which identify self-MHC I molecules) receptors, which either permit or restrain the killing capacity of NK cells, respectively. NK cells expanded *in vitro* recognize tumor antigen UL16-binding protein 2/5/6 on anaplastic thyroid carcinoma cells via natural killer group 2, member D receptor (NKG2D), thereby directing NK-mediated tumor lysis [47]. Conversely, self-MHC I expression protects cancer cells from death induced by licensed NK cells. Consequently, studies using patient samples demonstrate that cancer cells that down-regulate MHC I expression to evade T cell mediated cytotoxicity [[48], [49], [50], [51], [52]] become susceptible to NK cell-mediated cell death. Other immune cells can modulate the NK response when the innate immune response is activated. *In vivo* CXCR4 blockade on neutrophils or up-regulated NLRP3 inflammasome signaling in kupffer cells promotes IL-18 secretion, which in turn permits NK-cell licensing and enables FasL-mediated malignant melanoma cell elimination [53] and prevents metastatic growth of colorectal cancer cells in the liver [54]. CD16-stimulated NK cells require ROS for calcineurin/NFAT activation, which induces downstream FasL expression [55]. Unlike macrophages and neutrophils, licensed NK cells have a clear role in anti-tumoral immunity by targeting tumor cells for lysis.

To protect themselves from NK-mediated killing, cancer cells release immunosuppressive soluble factors into the TME. For example, acute myeloid leukemia cancer cells secrete soluble mediators that activate the aryl hydrocarbon receptor pathway in NK cells and directly impair NK-cell maturation and function, with studies using patient tissues showing that this is mediated through inducing the expression of miR-29b [56]. CXCL10 secreted by anaplastic thyroid carcinoma cells promotes CXCR3-expressing NK-cell migration into the TME. These NK cells that embed in the tumor stroma down-regulate NKG2D expression and exhibit a suppressed cytotoxic phenotype compared to peripheral NK cells [47]. One study showed that circulating NK cells recruited to the tumor stroma express atypical chemokine receptor 2 (CCR2), which suppresses CCR2 expression in KLRG1-expressing NK cells and limits their movement within the lungs towards other metastatic deposits [57]. Prostaglandin E2 secreted by thyroid cancer cells inhibits expression of the NK-cell-activating receptors NKp44 and NKp30 and death receptor tumor necrosis factor-related apoptosis-inducing ligand (TRAIL), resulting in suppressed NK cytotoxic function [47,58]. Even by-products of glycolysis produced by cancer cells, such as lactic acid, can impair NK-cell activity *in vitro* [59]. In response to an NK-cell attack, breast cancer cells remodel their actin cytoskeleton *in vitro* to increase expression of inhibitory ligands thereby signaling a dampened NK-cell lytic response [60]. As such, a presence of NK cells *per se* does not indicate tumor-cell elimination due to the possibility of a tumor-induced suppressed NK-cell profile.

### 2.4 T cells

Compared to cells of the innate immune system, cells in the adaptive immune system take time to respond to threats because the response is customized to the antigen. T cells comprise one of the major components of the adaptive immune response. In the context of cancer, there are two antagonistic classes of T cells that have important roles in the fight against cancer — cytotoxic CD8^+^ T cells and CD4^+^ regulatory T cells (i.e. Tregs).

#### 2.4.1 Cytotoxic CD8^+^ T cells

Cytotoxic CD8^+^ T cells are essential for direct killing of pathogens or transformed cells. Under physiologic conditions, naïve CD8^+^ T cells circulate within the periphery. Upon TCR-pMHC engagement, these naïve cells are rapidly activated, proliferate and differentiate into cytotoxic T cells. These effector CD8^+^ T cells bind to antigen-expressing target cells and release cytotoxins, such as perforin and granzyme B (GZMB), to induce target cell lysis.

Retrospective studies of ovarian and colorectal cancers have revealed a strong correlation between the presence of tumor-infiltrating T lymphocytes (TILs) and cancer survival [[61], [62], [63], [64]], suggesting that the presence of cytotoxic T cells in the tumor is crucial to clinical outcome. A substantial population of TILs are CD103-expressing tissue resident memory T cells [[65], [66], [67], [68]] that express numerous genes involved in cytotoxic function such as *IFNG, GZMB* and *CCL3* [69]. Besides being an adhesion molecule, CD103 is also vital for lytic granule exocytosis [70,71], cytokine production [70] and T cell recruitment into TGFβ-rich tumor regions [72]. Consequently, CD103-expressing CD8^+^ T cells have emerged as a prognostic marker for patient survival in lung, ovarian and bladder cancers [65,67,73,74].

The presence of competent cytotoxic CD8^+^ T cells in tumor regions does not guarantee total elimination of tumor cells because of a constant interplay between tumor and CD8^+^ T cells. These cells interact through three phases of cancer “immunoediting”. Phase 1 describes immune-elimination, where T cells kill off cancer cells. Phase 2 describes immune equilibrium, where surviving tumor cells co-exist with the anti-tumor immune cells. Finally, phase 3 describes immune escape, where surviving tumor cells overcome the immune control and progress to metastasis [75]. Cancer treatment thus encounters many challenges as tumor cells either adopt immune-evasive machinery to avoid recognition or create a non-conducive environment to inhibit an effective immune response.

As a safety mechanism to limit immunopathology, effector CD8^+^ T cells can become exhausted or undergo apoptosis upon long-term antigen exposure. Again, cancer cells outsmart the immune system and can exploit this host protective machinery to their advantage. Cancer cells with inherent genetic instability generate neoantigens. Prolonged exposure of T cells to an abundance of cognate antigens [76] induces signaling that elevates the expression of inhibitory molecules, such as cytotoxic T-lymphocyte protein 4 (CTLA-4) and programmed death 1 (PD-1) [77]. CTLA4 binds strongly to CD28 and obstructs co-stimulation signaling for T cell activation. As such, the T cell activation threshold increases and weak antigens, such as tumor antigens, cannot induce T cell activation. On other hand, the interaction between PD-1 and programmed death-ligand 1/2 (PD-L1/2) suppresses effector function [78] and promotes T cell apoptosis [79]. Samples obtained from patients with hepatocellular carcinoma show high PD1 expression on CD8^+^ T cells; in response to anti-CD3, these cells secrete less pro-inflammatory cytokines compared to low PD-1 expressing CD8^+^ T cells [80]. Similarly, in cervical cancer samples of patients that have progressed to the final stages of metastasis, PD-1 and PD-L1 expression is usually high [77]. However, chemotherapy further promotes PD-1 expression on CD8^+^ T cells [81], suggesting that PD-1-mediated T cell inhibition might underlie a failed response to chemotherapy.

To rapidly proliferate and acquire effector functions, activated CD8^+^ T cells rely heavily on aerobic glycolysis as a quick source of energy [82]. Tumor cells also predominantly utilize this metabolic pathway for energy, and thus compete with CD8^+^ T cells for glucose. A glucose-poor TME inhibits the up-regulation of phosphoenolpyruvate, which controls Ca^2+^-NFAT-mediated effector functions, resulting in T cell suppression [83]. In addition, glycolytic enzymes like GAPDH doubles up as mRNA-binding proteins when not engaged in glycolysis and have been shown to bind IFNγ mRNA, thereby preventing effective IFNγ translation [84]. Together, glucose-deprivation led to a less pro-inflammatory response by CD8^+^ T cells. Further, substantial aerobic glycolysis in the TME creates a harsh stroma that is lacking in oxygen and is rich in toxic metabolites. Tumor acidosis created by lactic acid accumulation impairs TCR co-receptor expression, inducing T cell anergy [85]. As seen in a melanoma mouse model, tumor-derived lactic acid also prevents NFAT upregulation, leading to diminish IFNγ production [59]. In the clinical setting, high levels of serum lactate dehydrogenase [86,87] or lactic acid [88] predicts poor prognosis in cancer patients. Unlike lactic acid, hypoxia does not suppress CD8^+^ T cell activity but instead promotes T cell survival and enhances T cell-mediated tumor control. It should be noted, however, that these superior effector functions are GLUT-1-dependent [89] and are, therefore, susceptible to glucose availability which is likely depleted in the TME. Altogether, tumor cells can suppress T cell effector activity through multiple avenues. First, an overwhelming interaction between tumor cells and T cells promotes intrinsic checkpoint expression. Second, a hostile extrinsic TME inhibits T cell survival and function.

#### 2.4.2 Regulatory T cells

Tregs are immunosuppressive cells with a central role in maintaining self-tolerance and immune homeostasis [90,91]. Like CD8^+^ T cells, Tregs also infiltrate the tumor stroma and a low CD8^+^ T cells to Tregs ratio is a poor indicator of disease outcome, overall survival and treatment outcomes in ovarian [92], breast [93] and bladder cancers [94]. Recently, Shabaneh and colleagues demonstrated that oncogenic BRAF_V600E_ in melanocytes drives Treg recruitment during the early stages of tumorigenesis in a melanoma mouse model [95]. Expression of CTLA4 on Tregs blocks T cells activation. Also, Tregs express constitutively high levels of IL-2 receptors (CD25, CD132). These receptors strongly bind IL-2, a cytokine also essential for CD8^+^ T cell proliferation and differentiation. Consequently, the presence of Tregs results in serum IL-2 consumption, limiting naïve T cell effector differentiation [96]. Further, Tregs can indirectly hamper CD8^+^ T cell activation by restraining expansion and immunogenicity of tumor-associated dendritic cells (DCs), leading to reduce IFNγ secretion and poor tumor control, as seen in an orthotopic pancreatic cancer model [97]. Surface-bound TGF-β on Tregs also suppresses cytotoxic T cell effector functions; blocking TGF-β with monoclonal antibodies restores T cell-mediated killing of tumor cells [98]. Although Tregs are generally pro-tumoral, some studies have revealed that the presence of tumor-infiltrating Tregs predicts a favourable prognosis in colorectal cancer [99,100] and Hodgkin's lymphoma [101]. This paradox could be due to the heterogeneous Treg populations [102] in each tumor site. Therefore, the exact role of Tregs needs to be carefully evaluated in each cancer type.

### 2.5 B cells

B cells can have either tumor-promoting or tumor-suppressive properties, depending on their subtypes, and are thus increasingly viewed as having a crucial role in cancer [103,104]. Through their intrinsic ability to recognize antigens and to regulate antigen presentation, B cells influence the activity of immune cells that express Fc receptors [104,105]. As the TME consists of a heterogeneous population of functionally distinct immune cells, the balance of various cell-specific responses indicates whether the B-cell population is poised for pro-tumorigenic or anti-tumorigenic functions.

Studies in mice and humans have identified discrete subsets of regulatory B cells (Bregs) that enable cancer cells to escape immune surveillance. Through elevated secretion of anti-inflammatory factors, such as TGF-β and IL-10 [106], Bregs can maintain immune tolerance and suppress both autoimmune and inflammatory responses. In addition to immunosuppressive cytokine secretion, Bregs suppress effector T cells and NK cells by expressing immune checkpoints, such as PD-L1 [107], and can promote metastasis by converting resting CD4^+^ T cells into Tregs [108]. To develop new anti-cancer strategies, unique Breg subsets need to be defined along with the mechanisms that underlie their activity. Zhang and colleagues identified a subset of CD5^+^ Bregs that bind IL-6 to give rise to phosphorylated STAT3 in murine models of melanoma and bladder cancer. Such signaling promoted tumor progression in prostate, ovarian and human non-small cell lung cancer (NSCLC) based on the patient tissues studied [109]. Finally, cross-talk between human as well as murine B cells and TAMs can favour M2 polarization in a PI3Kγ-depedendent manner, which supports tumor progression [110].

On the other end of the spectrum, there are B cell subsets that can exert an anti-tumor effect by serving as antigen-presenting cells (APCs) that contribute to the survival and proliferation of tumor-infiltrating T cells [111]. This function is especially relevant when DCs decline in activity or number, failing to sustain their role in presenting antigens to T cells. In murine models, B-cell depletion impairs CD4^+^ T cell activation and clonal expansion, suggesting that optimal antigen-specific T cell priming is achieved through the presence of B cells. A study of ovarian cancer patient samples found an association between prolonged survival and close proximity of CD8^+^ T cells to tumor-infiltrating B cells (TIBs) [112]. The presence of TIBs has also been linked to favourable clinical outcomes in NSCLC and breast cancer [113,114].

Other studies have shed light on the cytotoxic potential of B cells, where CpG-activated human B cells could kill cancer cells *in vitro* through TRAIL/Ap-2L-dependent mechanisms [115]. Treatment of leukemic cells with CpG *in vitro* also elevates GZMB levels and apoptosis in bystander B-chronic lymphocytic leukemia cells [116]. These findings thus highlight the potential possibility of B-cell-mediated immunotherapy to treat B-cell malignancies. One emerging therapeutic strategy involves isolating memory B cells from a human donor with a favourable cancer response, and propagating these cells to harvest the antibodies that they produce. The genes for selected antibodies can then be cloned into immortalized mammalian cells to enable the generation of an unlimited supply of tumorigenic antibody clones [103].

However, B-cell studies have been challenging due to a lack of common and robust phenotypic markers. This is further complicated by the up-regulation or down-regulation of these markers during immune activation [117]. This scenario has led to discrepancies in defining B-cell subsets depending on the experimental conditions; differences between humans and mice further add to an incomplete understanding of the role of B cells in cancer progression [105,118]. For instance, whether B cells (namely Bregs) actively promote tumor growth or whether an increase in Bregs simply reflects a natural immune response toward tumor cells is unknown. Nonetheless, immunotherapeutic strategies that aim to deplete, inhibit or strategically activate Bregs will make an invaluable contribution toward addressing cancer.

## 3 Cancer immunotherapy

### 3.1 Adoptive cell therapy

In recent years, cancer treatment has revolutionized to include immunotherapy as a new frontier. Immunotherapy utilizes the patient's immune system to attack the tumor of which adoptive cell transfer (ACT) is one of several immunotherapeutic approaches. ACT involves infusing either autologous or allogenic cells into patients. As the presence of intra-tumoral T cells is a positive prognostic marker for cancer survival [63,119,120], tumor-specific T cells make the perfect “live drug” for cancer therapy. Lymphokine-activated killer (LAK) cells are one of the earliest cell types used in ACT, where peripheral blood mononuclear cells (PBMCs) were cultured with IL-2 and the CD3 antibody clone OKT3. Comprised mainly of NK cells and NKT cells, LAKs have proven potent antitumor effects against various types of tumor cells in animal models and some clinical trials [[121], [122], [123]]. More recently, other strategies have been utilized to derive these tumor-specific T cells: (1) by isolating naturally occurring TILs from a resected tumor; (2) by generating cytotoxic T lymphocytes (CTLs) *ex vivo;* and (3) by engineering autologous T cells to express a tumor-specific chimeric antigen receptor (CAR). Each of these strategies is discussed in detail below.

*Tumor-infiltrating T cells* TILs are sourced from fresh resected tumor sections and expanded *ex vivo* to obtain large numbers for autologous infusion back into the patient. This technique enables harvesting of heterogeneous T cells with a large T cell repertoire with specificities to the tumor. Multiple TIL cultures can be derived from a single excised tumor biopsy and more importantly, each independent culture comprises a diverse phenotype (CD4^+^/CD8^+^ frequency) with antigenic specificities [124]. This approach was first used to treat patients with metastatic melanoma [125]. After a single dose of the chemotherapeutic cyclophosphamide, patients were intravenously injected with autologous TILs expanded *in vitro* followed by several doses of IL-2 to further promote T cell proliferation and function *in vivo*. This strategy achieved 55% objective cancer regression in multiple organs, but only 1 of 20 patients (5%) achieved complete regression [125]. Introducing a lymphodepletion regimen prior to ACT, either non-myeloablative chemotherapeutics or myeloablative total body irradiation, achieved durable complete metastatic melanoma regression in 22% of patients [126]. An independent clinical trial performed by another research group confirmed the benefits of non-myeloablation in ACT with a complete response in 15% of affected patients [127]. This preparative step prior to ACT can thus enhance the ACT response and have positive clinical outcomes.

A major challenge in using TILs is generating sufficient numbers of tumor-specific T cells that retain their killing capacity *in vivo*. Chacon et al. overcame this obstacle by using agonistic anti-4-1BB/CD137 during the early stages of *ex vivo* T cell expansion. Here, they achieved enhanced T cell yield (by 20%), activation signal (CD28) expression and anti-tumor activity [128]. A high proliferative response was achieved upon re-stimulation with tumor-specific antigen *in vitro* [128], and activation-induced T cell death was prevented [129]. The overall result was significantly improved T cell persistence *in vivo* after ACT. Another limitation of using TILs for ACT is that only patients with sufficient TILs are eligible for this treatment option. For example, the current success of TIL infusion in metastatic melanoma hinges on the fact that >80% tumor explants generate bulk TILs sufficient for ACT [124,127,130]. Other cancers, such as renal cell carcinoma and breast cancer generate fewer TILs and thus achieve a lower success rate [131,132]. This caveat greatly restricts the application of TILs infusion in cancer treatment.

*Cytotoxic T cells* CTLs are generated by stimulating autologous peripheral blood-derived CD8^+^ T cells with autologous DCs pulsed with known tumor antigens [[133], [134], [135]]. These CTLs produce an anti-tumor response characterized by IFNγ secretion [[135], [136], [137], [138]] and exhibit antigen-specific killing of target cells [[133], [134], [135], [136]]. The clinical outcomes after CTL cancer treatment are varied. Patients with progressive refractory metastatic melanoma showed an improved clinical response with better survival (11 months versus 4 months) after MART1/gp-100 specific CTL infusion [133]. MART1-specific CTL infusion induced a specific loss of MART1-expressing tumor cells [133]. This specific loss of MART1-expressing tumor cells was observed in another independent study of patients with metastatic melanoma [134]. The disappearance of MART1-specific cancer cells suggests that either these antigen-expressing tumor cells undergo on-target lysis by the infused CTLs or there is a problem in developing antigen escape variants. The latter is a point of concern as this effect renders MART1-specific ACT ineffective in the absence of MART1-expressing targets. This problem can be addressed by generating heterogeneous CTLs with a broader tumor-antigen-specific T cell repertoire, possibly by stimulating DCs pulsed with apoptotic tumor cells [137,138].

Adoptive transfer of gp100-specific CTLs into melanoma tumor-bearing mice induces massive infiltration of myeloid-derived suppressor cells (MDSCs) into the tumor, which suppresses anti-tumor responses over the long term [139]. We thus speculate that CTL infusion might impact ACT-induced recruitment of MDSCs into the tumor, which negates the anti-tumor activity of T cells.

*Chimeric antigen receptor T cells* CAR T cells were designed to overcome the limitation that CTLs only recognize antigens in an MHC-restricted manner. By this approach, T cells from either patient or donor, are collected and then genetically modified to express chimeric receptors specific to a tumor antigen, along with a CD3ξ signaling domain and co-stimulatory molecules. This fusion of the antibody-derived single chain variable fragment with the T cell intracellular signaling domains endows the CAR T cell with the ability to recognize the tumor antigen in a non-MHC-restricted manner [140]. This approach thus overcomes the issue of spontaneous loss of MHC class I expression on tumor cells.

Success of CAR T therapy is evident in hematologic malignancies with the eminent FDA approval of Kymriah™ (Tisagenenlecleucel, Novartis, USA) for acute lymphoblastic leukemia (for patients up to 25-years old) and diffuse large B-cell lymphoma, and Yescarta™ (Axicabtagene Ciloleucel, Kite Pharma, US) for large B-cell lymphoma. Both FDA-approved CAR T cell products target CD19 that is ubiquitously expressed on B cells but not on bone marrow stem cells or other tissues. During the phase 2 clinical trials, Kymriah reported an 81% overall remission rate within 3 months [81] and Yescarta reported a 54% complete response rate [141]. From the perspective of cancer intervention, these statistics demonstrate unprecedented clinical success.

While there are already two FDA-approved CAR T treatment options for hematologic malignancies, CAR T therapy has encountered hurdles in the treatment of solid tumors due to the added complexity of the TME. Here, tumor antigen expression is heterogeneous in terms of intensity and distribution [[142], [143], [144]]. As such, CAR T treatment on solid tumors encounters safety concerns such as “on-target, off-tumor” toxicity where CAR T cells target normal cells that express tumor-associated antigens [145,146]. To circumvent this problem, Kloss et al. [147], and Zhang et al. [148], designed dual-antigen specific CAR T cells for a pancreatic and prostate cancer cell line, respectively. In this modified approach, two tumor-specific antigens are selected and each antigen fuses with a signaling domain such that there are separate tandem constructs of antigen1-CD3ξ and antigen2-4/1BB on each CAR T cell. This physically separates signaling structure and confines persistent T cell function to only cognate target cells that express directed antigens; no cytotoxicity against single-antigen expressing cells is produced [148], thus ensuring therapeutic accuracy.

Another strategy to counteract “on-target, off-tumor” toxicity is through the use of switchable CAR T cells. Here, T cell activation only occurs in the presence of a Fab switch against both tumor antigen and a peptide neoepitope [149]. Switchable human epidermal growth factor receptor 2 (HER2) CAR T cells were found to be as effective in tumor control as conventional HER2 CAR T therapy, in an orthotopic model of advanced pancreatic ductal adenocarcinoma (PDAC) and in a patient-derived PDAC xenograft mouse model. Switchable HER2 CAR T therapy, however, affords a tunable response that protects healthy tissues that express some tumor antigens [149].

Another key factor that hinders effective ACT in solid tumors is the immunosuppressive TME. Interestingly, CAR T cell infusion can also transform the immunosuppressive TME into an immunostimulatory environment [150,151]. For example, CAR T therapy specific for glioma antigen resulted in pro-inflammatory cytokine secretion and an altered immune-cell landscape, with an increase in CD8^+^ T cells and a decrease in MDSCs in tumor-bearing mice grafted with a human glioma cell line [150]. A more direct avenue to establish a pro-inflammatory TME is through infusion of T cells re-directed for universal cytokine-mediated killing (TRUCKs) [152]. Instead of stimulating CAR T cell activation and cytotoxic killing, an *in vitro* study demonstrated that TRUCKs release pro-inflammatory IL-18 upon CAR engagement with a cognate tumor antigen. IL-18 then polarizes T cells towards T-bet^high^ Foxo^low^ effector cells with sustained cytotoxic function, preventing T cell exhaustion. At the same time, TRUCKs can skew the TME to be less pro-tumoral, by reducing the number of M2 macrophages and Tregs, further relieving T cell suppression, thus improving the survival of mice with advanced pancreatic and lung tumors [152]. Finally, by genetic engineering, CAR T cells which were manipulated to specifically target human colony-stimulating factor 1 receptor (CSF1R) could control pro-tumoral M2 macrophage differentiation, thereby removing their T cell suppression effect *in vitro* [153]. Taken together, there are numerous possibilities to engineer CAR T cells to generate an effective strategy against solid tumors. Developments are now only limited by safety issues of the construct.

*Chimeric antigen receptor NK cells* NK cells are another subset of cytotoxic cells responsible for killing damaged cells. As such, they are also excellent targets to drive tumor cell-specific cytolysis. An added advantage of NK cells over T cells is their short life span of only 2 weeks [154] thus limiting “on-target, off-tumor” adverse effects. Compared to T cells, NK cells can be obtained from multiple sources, such as peripheral or umbilical cord blood and derived from human embryonic stem cells or induced pluripotent stem cells (iPSCs). In the field of bioengineering, NK cell lines, such as NK-92, are favored over primary human NK cells as they have almost no expression of inhibitory killer cell immunoglobulin-like receptors and yet display cytotoxicity that is equivalent to activated NK cells even upon irradiation [155]. Using a similar design strategy as CAR T cells, CAR NK-92 cells have demonstrated their efficacy against tumors, with these engineered NK cells currently undergoing clinical trials for hematologic malignancy (NCT02892695, NCT02742727 and NCT02944162) and solid tumors (NCT02839954). To ease reliance on IL-2 for expansion, an IL-2-independent NK-92MI cell line has been derived from the NK-92 line. This newly derived NK cell line can be efficiently transfected and displays similar characteristics to the parental NK-92 cells [155]. Most importantly, these cells kill cognate tumor cells and their cytotoxic capacity is associated with tumor antigen expression on target cells [156]. Induced expression of tumor antigen by epigenetic modifiers, such as sodium butyrate or DNA methylation inhibitor, as in the case of carcinoembryonic antigen in human colorectal cancer cells, promotes CAR NK-92MI-mediated cytotoxicity [156].

Besides degranulation upon antigen recognition, NK cells also participate in antibody-dependent cell-mediated cytotoxicity [157]. Making use of the Fc-mediated machinery, Chen et al., modified NK-92MI cells to express the Fcγ receptors CD16 or CD64, and showed that NK-92MI cells expressing these Fcγ receptors selectively killed CD20-expressing tumor cells in the presence of anti-CD20 [158]. Because the practice of therapeutic antibodies is well established [159], engaging CAR NK cells will definitely boost treatment outcomes. Transforming the TME can also be achieved by NK-92MI cells, targeting CSF1R resulting in the elimination of CSF1R-expressing pro-tumoral M2 macrophages [153]. The typical CAR design for NK cells uses the same signaling domain (CD3ξ) and co-stimulatory molecules (CD28/CD137 (4-1BB)) as CAR T cells. This design disregards inherent NK-cell signaling. Genetically modified NK-92 cells expressing NKG2D, a key activation receptor of NK cells, have higher anti-tumor activity and CD107 expression (marker for degranulation) than NK-92 cells expressing a CAR T construct (CD28^−^CD28^−^CD137-CD3ξ), highlighting the importance of customized signaling molecules in the design of CAR NK cells for optimal NK-cell activation and function [160]. Finally, iPSC-derived CAR NK cells are also effective in controlling tumor growth, as shown in a mouse xenograft ovarian cancer model [160]. Compared to CAR T cells, which also display a potent ability to reduce tumor burden, CAR NK cells induce fewer adverse effects, such as weight loss and organ damage, and prolong survival in treated mice [160]. These findings suggest that CAR NK cells may be a safer option than CAR T cells.

**Fig. 2:**
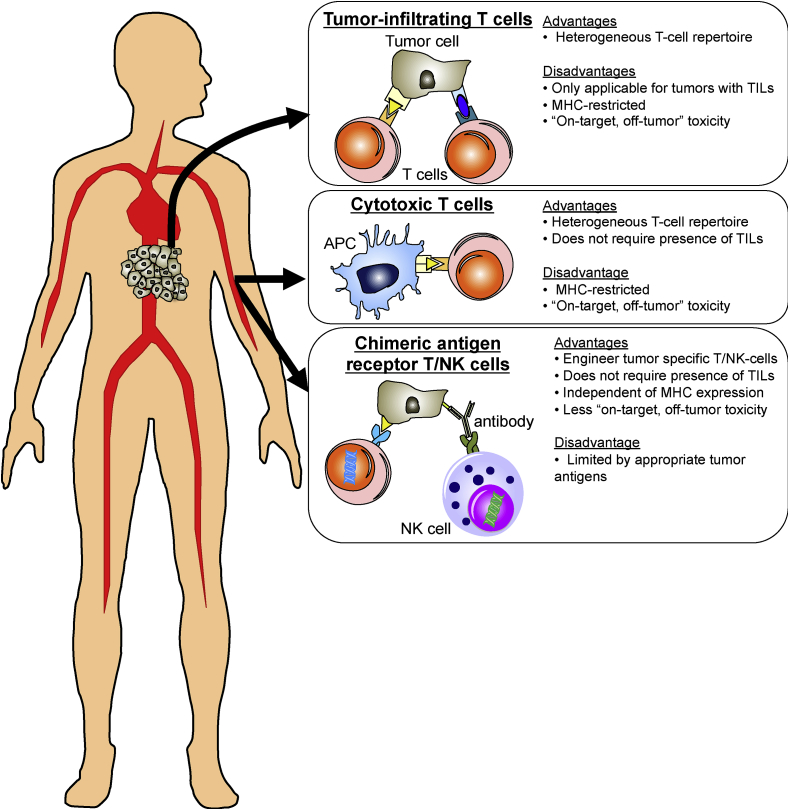
**Advantages and disadvantages of each strategy of adoptive cell transfer.** Tumor-specific T/NK-cells can be derived using three ways, namely from tumor (tumor-infiltrating T cells (TILs)), cultured from naïve T cells (cytotoxic T cells) or genetically engineered (chimeric antigen receptor T cells). Harvest of TILs ensure heterogeneous T cells with a large repertoire. However, not all tumors have sufficient TILs for treatment. In vitro generation of tumor-specific T cells from peripheral T cells circumvent this limitation of TILs. Yet, both types of T cells rely on stable antigen-MHC expression. To overcome this, chimeric antigen receptor (CAR) T cells is designed. Besides, NK cells can also be genetically engineered to target tumor cells. NK CAR has less “on-target, off-tumor” toxicity due to the short lifespan.

Fig. 2 summarizes the advantages and disadvantages of each strategy of ACT. Of note, one major advantage of TILs is that they are enriched for tumor-specific T cells. Furthermore, the use of TILs avoids the disadvantage of high cost in producing CAR T and CAR NK cells. Today, the application of ACT to cancer has moved beyond the challenges of clinical translation. Now, the biggest hurdle is to identify appropriate antigen targets that will maximize anti-cancer responses with minimal adverse effects. In this respect, advances in data science that predict neoepitopes and patient transcriptome analyses may help identify and select suitable candidate targets.

### 3.2 Immune checkpoint inhibitors

Immune checkpoints collectively refer to a set of co-stimulatory, such as CD28, and co-inhibitory, namely CTLA-4, PD-1, TIM-3 and LAG-3, signals that are necessary for immune homeostasis and host survival. A balance between these signals allows for self-tolerance under normal physiological conditions and protects the host from tissue damage during an immune response against a foreign antigen [161]. As activated T cells are the primary mediators of immune effector functions, they strongly present with multiple co-inhibitory receptors such as PD-1 and CTLA-4 [162]. In the event of malignancy, immune checkpoint molecules are co-opted, preventing effector T cells from mounting an effective anti-tumor response [163].

Clinically, immune checkpoint blockade (ICB) has been exhibited promising data across numerous solid tumor types (including bladder [164], breast [165,166], colorectal [165,[167], [168], [169], [170]], gastric [165], ovarian [165,166], pancreatic [165,166], prostate [167,170], uterine [166], non-small-cell lung cancer (NSCLC) [[165], [166], [167],[170], [171], [172], [173]], head and neck squamous cell carcinoma (HNSCC) [166], renal cell carcinoma (RCC) [[165], [166], [167],^170^,^174^], sarcoma [166], and melanoma [[165], [166], [167],^169^,^170^,[175], [176], [177], [178], [179], [180], [181], [182]]), as well as hematologic malignancies (including diffuse large B-cell [183], follicular [184], and Hodgkin lymphoma [185]). Accordingly, ICB is being implemented in an expanding array of cancer clinical trials, with four ICB agents already approved for clinical practice [160]. However, therapeutic efficacy remains broad across patients and cancer types, with only a subset of patients showing a durable response [160,186]. Amongst the various cancer types, current clinical data suggests that bladder cancer [164], melanoma [[165], [166], [167],^169^,^170^,[175], [176], [177], [178], [179], [180], [181], [182]], mismatch repair–deficient colorectal cancer [168], and certain hematopoietic malignancies [183,185] are the most responsive to ICB, and the trajectory of patient response across different cancers has been extensively reviewed elsewhere and will not be further elaborated in this review.

Initial clinical research in mice revealed that antibody blockade of CTLA-4 elicits a successful anti-tumor response [187,188], placing CTLA-4 as the first immune checkpoint molecule to be clinically targeted. PD-L1/PD-1 was later discovered and subsequently targeted. Compared to PD-1 and CTLA-4, less is known regarding the signaling mechanisms associated with lymphocyte-activation gene 3 (LAG-3) [[189], [190], [191]], killer immunoglobulin-like receptors (KIRs) [192,193] and T cell immunoglobulin mucin 3 (TIM-3) [[194], [195], [196]]. However, in patients with melanoma, a reverse in tumor-induced T cell exhaustion/dysfunction was achieved via dual blockade of TIM-3 and PD-1, which restored T cell secretion of IFNγ and TNFα [197].

Studies in pre-clinical *in vitro* and *in vivo* cancer models have hence demonstrated that blocking multiple checkpoints with specific monoclonal antibodies can, therefore, result in improved survival outcomes [[198], [199], [200], [201]]. Moreover, the co-expression of multiple immune checkpoints in patients frequently correlates with increased T cell suppression [202,203]. These findings suggest that individual checkpoint molecules are predominantly governed by non-overlapping molecular mechanisms [204]. This concept is further supported by pathway analysis of blood and tissue samples from patients undergoing single or combined ICB, in which distinct genomic and functional signatures were observed after specifically blocking CTLA-4 and/or PD-L1 [205]. Therefore, as both CTLA-4-based and PD-1-based mechanisms act on different aspects of T cell suppression, there is strong rationale to also combine PD-1 and CTLA-4 blockers to achieve enhanced therapeutic outcomes. In fact, several preclinical murine studies have shown that combined anti-PD-1/CTLA-4 regimes decrease tumor progression and prolong survival [206,207].

In the clinic, combinational therapy has improved clinical responses in melanoma patients and as such, these treatments have been approved as a first-line therapy for patients with advanced melanoma [176,182,205,[208], [209], [210]]. Clinical trials have also demonstrated that substantial improvements in disease-free survival can be achieved in patients with Hodgkin lymphoma and metastatic lung cancer [172,184,[211], [212], [213]]. Such progress affords hope toward achieving the primary goal of oncotherapy, which is to reinstate immunological control of tumor growth.

The overall success of ICB hinges on identifying predictive biomarkers or hallmarks of a response, especially in the long term, to checkpoint blockade. For example, PD-L1 expression by tumor and/or antigen-presenting cells (APCs), particularly macrophages and myeloid dendritic cells, has been correlated with increased patient response toward anti-PD-L1/PD-1 therapy in patients with colorectal cancer [214], HNSCC [215], melanoma [165,170], NSCLC [172,215] and RCC [214]. In patients with metastatic NSCLC, pembrolizumab is approved in conjunction with the PD-L1 IHC 22C3 pharmDx assay (Dako), a companion diagnostic test that identifies suitable patients for pembrolizumab therapy [216]. A correlation has also been observed between tumor neoantigen load (i.e. tumor neoantigen mutation burden) and the cytolytic activity of CD8^+^ T cells [217]. Finally, the accumulation of TILs in the TME has been associated with prognosis and clinical response following neoadjuvant therapy in rectal [218], and breast cancer [[219], [220], [221]].

The pursuit for improved biomarkers has prompted research groups to develop improved scoring strategies that take into account a patient's pre-existing immunity for predicting disease-free survival. Conceptually, these scoring strategies consider traditional tumor node metastasis staging together with the “immune contexture” of a patient, to create a holistic snapshot of the tumor-immune profile to predict a clinical response. Pagès and colleagues coined the term “immune score”, and asserted that the type, density and location of the immune infiltrate at the TME serves as a novel prognostic factor to predict disease-free survival in addition to histopathological parameters. They propose that using the “immune score” will aid decision-making regarding adjuvant therapies in early-stage cancers [222]. Such predictions must account for plausible patient-specific differences in the relative role of different immune checkpoints and how these roles may evolve (a subject area in need of further research). Indeed, an extensive study of T cell expression of multiple checkpoint proteins found that naïve T cells are controlled by TIM-3 and BTLA, while tumor-infiltrating effector T cells express a wider range of checkpoint molecules depending, to a certain degree, on their anatomical location [162]. Others have shown that compensatory mechanisms of immune suppression can follow PD-1 blockade, whereby T cells up-regulate TIM-3 expression after exposure to anti-PD-1 treatments.

Henceforth, a paradigm shift in future ICB therapy may be underway, where both a patient's pre-existing immunity as well as ensuing an immune response may be used as a foundation to identify a suitable combination course of ICB for treatment. Risk of adverse events such as toxicity of the administered checkpoint blockade is, however, an important consideration, with inflammatory immune-related adverse effects being most apparent in previous clinical trials [211,223]. While most effects are reversible, death from myocarditis pneumonitis, colitis and neurologic events, among others, can occur [224]. Therefore, in addition to identifying response-predictive biomarkers and developing strategies that can better-inform diagnosis and recommendations of ICB, vigorous methods to assess such adverse events must be enforced.

### 3.3 Immunometabolism

One hallmark of cancer is the reprogramming of cellular metabolism [2]. For cancer cells to survive and thrive in a hypoxic microenvironment, they must reprogram their metabolic profiles and energy requirements to fuel their cellular outputs and adapt to the TME [225]. One of the main metabolic pathways utilized by cancer cells to permit rapid fluctuations in energy demands is known as the Warburg effect, whereby cells preferentially undergo glycolysis even in the presence of oxygen [226].

There has been burgeoning interest in the field of immunometabolism and its impact on disease pathogenesis [18,227,228]. Immunometabolism describes the interplay between immunologic and metabolic processes, where the immune system mediates cancer initiation and development. Immune cells use and respond to nutrients in a similar way to other cells. Recent *in vitro* and *in vivo* studies have also shown that activated immune cells also exhibit specific metabolic profiles that direct their downstream effector functions [229,230]. T cell activation, survival and function following transition from a naïve T cell to effector and a memory T cells, is dependent on the cellular metabolic profile at each phenotypic stage [231]. Activated T cells that have differentiated from naïve T cells require ATP to support rapid cell growth. This metabolic reprogramming involves the switch from oxidative phosphorylation to glycolysis with available nutrients, such as glucose and glutamine in the environment [232]. Furthermore, each CD4^+^ T effector and regulatory cell subset has a unique metabolic phenotype correlating to its Th1, Th2 or Th17 effector functions [233]. Modulating immunometabolism and targeting glucose metabolism can be an effective method to control cell-fate determination and reprogram downstream effector functions. In a murine model of experimental autoimmune encephalomyelitis (EAE), treatment with the glycolysis inhibitor 2-deoxyglucose (2-DG), successfully suppressed EAE by dampening T cell development into Th17 cells and promoting Treg generation [201]. Furthermore, activated T cells in patients with allergic asthma produce high levels of lactate correlating to an upregulation of pyruvate dehydrogenase kinase-1 (PDK-1) [234]. Isolation of activated CD4^+^ T cells from asthma patients followed by treatment with the PDK-1 inhibitor dichloroacetate (DCA) inhibited T cell proliferation and reduced cytokine production by promoting oxidative phosphorylation instead of glycolysis [234].

In the context of regulating treatment responses, TAMs secrete cysteine cathepsin proteases that protect tumor cells from destruction: this process blunts the chemotherapeutic response in patients with breast cancer [235]. Targeting TAMs by inhibiting colony-stimulating factor-1 receptor (CSF1R) or chemokine (C–C motif) receptor 2 (CCR2) decreases the number of pancreatic tumor cells and improves chemotherapeutic efficacy, inhibits metastasis and increases anti-tumoral T cell responses *in vivo* [236]. Strategies that effectively target TAMs and/or repolarize TAMs towards an anti-tumoral phenotype, therefore, will help eliminate tumor cells.

Our group previously showed that *in vitro*-tumor conditioned macrophages exhibit a pro-metastatic phenotype, with a capacity to promote angiogenesis, epithelial-mesenchymal transition (EMT) and extravasation to facilitate tumor dissemination. In parallel, we also observed that these macrophages show an elevated glycolysis rate, which is a characteristic of the Warburg effect. Notably, inhibiting glycolysis using 2-DG inhibited the macrophage pro-metastatic phenotype, reversing their angiogenesis, extravasation, and EMT capabilities [18]. Others have also reported that changes in the metabolic strategy used by TAMs are linked to tumor invasion, angiogenesis and metastasis [237,238].

It is now appreciated that cell-intrinsic metabolism directly controls effector function and cellular fate. These metabolic programs are controlled, in part, by the phosphatidylinositol-3-kinase (PI3K)/Akt/mammalian target of rapamycin (mTOR) signaling pathway [239,240]. For example, in a mouse model of arthritis, inhibiting both the mTOR and MAPK pathways with rapamycin and a MEK1/2 inhibitor, PD325901, effectively inhibited effector CD4^+^ T cell activation [241]. As such, anti-inflammatory agents targeting PI3K, Akt, and mTOR may be a viable option to treat inflammatory-driven diseases. Targeting immune-cell metabolism may provide an opportunity to modulate the balance between anti-inflammatory and pro-inflammatory, effector and regulatory immune responses. Modulating immunometabolism for immunotherapy may, therefore, provide new directions to treat an array of infections, inflammatory diseases, and ultimately cancer.

### 3.4 Nano-immunotherapy

Advances in immunotherapy have given rise to a wealth of new and promising therapeutic modalities, which include cellular therapies, monoclonal antibodies, small molecules, proteins and peptides. However, the clinical benefit of all these modalities is limited by delivery challenges which include, but are not limited to, nonspecific uptake by phagocytic cells, poor target specificity, poor permeation through tumor tissue, and off-target bio-distribution [242]. Strategies must, therefore, be developed that allow immunotherapeutics be delivered with appropriate kinetics and distribution while avoiding any undesirable adverse effects that would offset the clinical benefit to a patient.

Nanoparticles (NPs) have emerged as a versatile solution to the therapeutic constraints described above, due to their favourable transport properties, biodistribution behaviour and unique surface properties for functionalization [243]. With the rapid development of biomaterials that respond to various stimuli (including pH, temperature and electrical charge), sophisticated systems of multifunctional NP systems are being developed that are anticipated to expand the therapeutic possibilities afforded by NPs [[244], [245], [246], [247], [248]]. The use of engineered NPs for therapeutic purposes in cancer is experiencing a period of pronounced development, offering the promise of more efficient and specific delivery of therapeutic cargos to secluded targets in the TME. Several cancer nanotherapeutics have been approved by the FDA to date [249]. However, FDA-approved options are based on the delivery of chemotherapeutic drugs aimed toward the malignant tumor, with no purposed effect on the immune cells of the TME. With a growing appreciation of the crucial role of immune cells in cancer (as highlighted in the earlier sections), the focus of nanotherapeutics is thus shifting toward modulating the activity of immune cells for anti-cancer treatment.

*Nanoparticle properties and biological identity* Tuning the physicochemical properties of NPs defines the extent by which they attach to, are internalized by, or are intracellularly trafficked in an immune cell [250]. A key concept to appreciate in designing NPs for immunotherapy is that ultimately, it is the physicochemical properties *in vivo* that determine the interactions of NPs with immune cells. More specifically, when NPs are exposed to blood or lymph fluid, plasma proteins rapidly adsorb and deposit on the NP surface, forming what is commonly referred to as a protein corona [251,252]. The corona's composition differs depending on the intrinsic physicochemical properties of the NP [253]. Therefore, *ex vivo* physicochemical characterizations of NPs cannot be simply extrapolated to the eventual properties of NPs *in vivo*, and caution should be exercised when using these *ex vivo* characterizations to predict the *in vivo* interactions of NPs with immune cells.

Data suggest that the protein corona is dynamic, whereby the exact composition of proteins on the NP surface evolves over time [254,255]. This concept supports the notion of designing NPs that make use of a recognition hierarchy to either target different cells over time, or temporally modulate cell activity via the evolving corona. Such a scenario was previously described in the context of different macrophage subtypes, which differ in their affinity for different opsonic proteins. The type of opsonic protein(s) on the NP surface and/or changes in surface opsonization processes are proposed to define the macrophage subpopulation that hosts the NP [250]. Further understanding of differential opsonization processes for different biomaterials would facilitate the design of NP carriers with high specificity towards immune-cell subtypes.

*NP interactions with macrophages* Macrophages and circulating monocytes (Mo) are major phagocytic cells with a powerful capacity to detect and uptake NPs. Such ability means that Mo and macrophages impede the target-specific delivery of drugs, but also positions them as vulnerable targets that can be strategically utilized in cancer treatments. Mo/macrophage-specific targeting can be facilitated by surface grafting NPs with suitable peptides or ligands, such as the family of scavenger receptors, Dectins and mannose receptors, Fc and complement receptors [256]. One mouse study showed that mannose-functionalized polymeric NPs co-entrap melanoma-associated antigens and Toll-like receptor (TLR) adjuvants. That study used Poly(I:C) and CpG (both known to potentiate a Th1 response) adjuvants targeted to mannose receptors on macrophages and other APCs [257]. This approach induced an anti-tumor immune response, as observed via elevated IL-2 and IFNγ secretion. Subsequently, the treated mice showed a marked delay in tumor growth [257].

The tumor-homing ability of Mo/macrophages renders them excellent candidates for cell-mediated delivery of therapeutic cargo. Here, macrophage-mediated delivery can be achieved by means of intracellular entrapment and subsequent release of cargo at the tumor site. Referring to the analogy of a cellular “Trojan Horse”, Choi and colleagues showed that Mo/macrophages that phagocytized gold nanoshells (Au-NS) could be recruited to a breast tumor spheroid using an *in vivo* mouse model. Subsequent cancer-cell death could be triggered by photo-induced ablation of Au-NS-loaded Mo/macrophages [258,259]. Alternatively, therapeutic cargo can be attached to the Mo/macrophage surface in the form of a nano-sized “cellular backpack”. This strategy was demonstrated *in vitro* by Doshi et al., whereby Mo-associated cellular backpacks were capable of releasing a model protein in a controlled and sustained manner [260]. Similar findings were demonstrated by Anselmo et al., who also showed that Mo-cellular backpacks could target and accumulate in the inflamed organs of the mouse model to a greater extent than “free” backpacks, and these backpacks exhibited low levels of accumulation in clearance organs, allowing for more sustained targeting [261].

A final NP-based approach that has gained renewed interest over recent years is based on re-programming TAMs from a pro-tumoral M2 to an anti-tumoral M1 phenotype. Castro et al. demonstrated the ability of chitosan/poly(γ-glutamic acid) NPs to successfully re-polarize IL-10-stimulated macrophages *in vitro* towards a pro-inflammatory profile, with decreased CD163 expression and increased TNF-α secretion [262]. In another study, low dose exposure to photodynamic sensitization using Temoporfin NPs triggered M1 re-polarization of (previously M2-polarized) THP-1 cells [263].

NPs that are meant to target immune cells other than Mo/Macrophages need to avoid Mo/macrophage clearance. To this aim, NPs can be coated with cell membrane components to form a sink for anti-red blood cell antibodies as demonstrated *in vivo*, thus preventing phagocytosis and destruction by macrophages [264]. Intriguingly, at the point of initial contact, the physical shape of NPs can also control whether macrophages proceed with phagocytosis or not as long as the NP volume does not exceed the volume of the cell. This phenomenon has led to the design of shape-shifting NPs using stimulus-responsive polymers, whereby shifting from a spherical to an elliptical disk shape minimizes phagocytosis of NPs by macrophages [265].

*NP interactions with T cells* T cells represent another major cell type of specific immunity, with a crucial and well-established role in ameliorating tumor cells. Given their central role in the immunological network, they have been a logical and attractive target in NP-based immunotherapeutic strategies [266,267]. The therapeutic potential of T cell–NP immunotherapy was revealed by Schmid and colleagues who achieved PD-1-targeted delivery of NPs to CD8^+^ TILs. The NPs delivered a TGF-β signaling inhibitor, which extended the survival of tumor-bearing mice. In the same study, delivery of a TLR 7/8 agonist increased the proportion of CD8^+^ T cells in the TME of the mouse model, which sensitized tumors to subsequent anti-PD-1 treatment. By contrast, free drugs administered at similar doses had no observable effect [268].

Similar to Mo/macrophages, T cells can also serve as cellular chaperones of therapeutic cargo. Siriwon and co-authors used CAR T cells to deliver an antagonist of T cell-immunosuppressive A2a adenosine. By taking advantage of the tumor-penetrating properties of T cells, the authors demonstrated using a mouse model that surface-engineered CAR T cells could effectively deliver the antagonist (adenosine receptor small molecule antagonist, SCH-58261) to deep regions of the immune suppressive TME for subsequent release [269]. NPs can also provide a functional boost to T cells to either restore or augment their anti-tumor activity [270]. Kosmides et al. developed platform for stepwise T cell activation, thus allowing for controlled and customized T cell stimulation. The researchers generated monospecific paramagnetic NPs by conjugating them with distinct single signal antigens, and then used a magnetic field to selectively cluster different NP-antigens *in vivo* to strategically activate T cells [271].

Nanotechnology has also been explored as a means to improve upon existing strategies of adoptive transfer of transgenic T cells, including CAR T cells [272,273] and T cells that are redirected to viral-associated tumors by means of a peptide-specific TCR [[274], [275], [276]]. Smith et al. developed a DNA-carrying NP that could efficiently deliver leukemia-specific CAR genes into T cell nuclei *in vitro*, resulting in the long-term disease remission of mice that were administered with the DNA-carrying NPs. This approach represents a practical alternative to the costly and time-consuming method of isolating and expanding tumor-specific T cells to re-infuse into patients at a sufficient dose. In this way, anti-tumor immunity is elicited “on-demand” to generate a sustained population of anti-tumor T cells [277].

*Personalizing NPs with tumor neoantigens* Moving forward, NP systems need to gear towards personalized immunotherapy [[278], [279], [280]]. To this end, identifying tumor neoantigens has rekindled scientific creativity in the design of patient-specific nanomedicines [281,282]. The concept of tumor neoantigens has its roots in cancer immunoediting, which attempts to explain the paradox of tumor formation in an immunocompetent host, where the immune system has a dual host-protective and tumor-promoting role [283]. This being the case, tumor neoantigens present as attractive targets for cancer therapy, as they modulate the immune response [284].

The therapeutic potential of neoantigens was observed in clinical studies, where patients received a potent yet highly compatible “therapeutic hit” against tumor growth [285,286]. Work by Zhu et al. showed that self-assembled, intertwining DNA–RNA nanocapsules could efficiently deliver synergistic DNA and RNA adjuvants and tumor neoantigens into APCs *in vitro*. Moreover, mice that were administered with nanocapsules displayed elevated levels of neoantigen-specific CD8^+^ T cells in the peripheral blood, with an inhibited progression of neoantigen-specific colorectal tumors [263]. Similarly, Qiu et al. created pH-responsive nanoplexes of tumor neoantigens that exhibited increased and prolonged antigen uptake by DCs for sustained surface presentation *in vitro*, which resulted in enhanced CD8^+^ T cell activation [287].

**Fig. 3:**
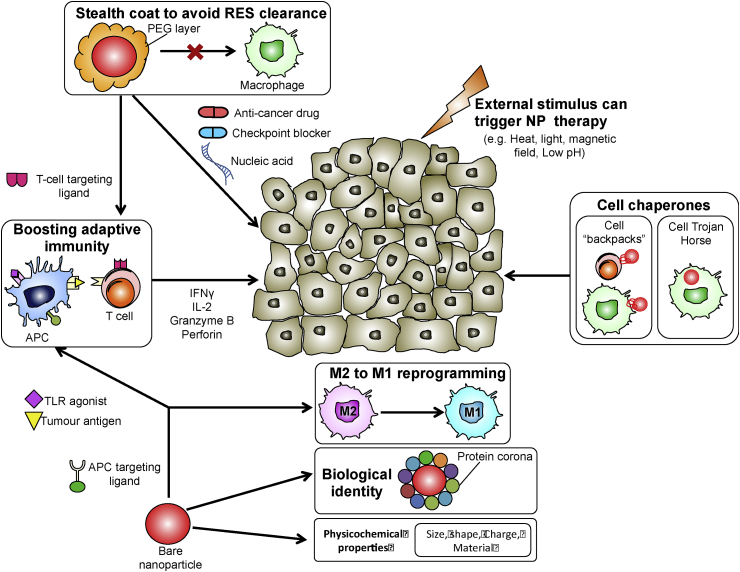
**Recent developments in nanoparticle (NP) – mediated immunotherapy may target antigen-presenting cells (APCs) or the adaptive arm of immunity.** Cell targeting ligands enable specific delivery to each cell type. To avoid non-specific uptake by the reticular endothelial system (RES), NPs may be conferred with stealth properties by modifying their surface with polyethylene glycol (PEG). Strategies of NP immunotherapy include boosting T cell effector function, M2-M1 reprogramming of macrophages, using immune cells as cellular chaperones of therapeutic cargo or NP-enhanced delivery of monotherapy or combination therapy. Stimuli (either external or by tumor environment conditions) may trigger NP release of therapeutic cargo. Interaction of NPs with immune cells depend on the NP's biological identity as defined by the protein corona, and its physicochemical properties.

*Testing NP immunotherapeutic potential* Relevant also to the development of NP for immunotherapy is the identification of a suitable treatment window. Degradable NPs can be developed to achieve a therapeutic delivery method with controlled kinetics. For example, biodegradable PLGA NPs can deliver TLR adjuvants at a slower and more controlled rate than TLR adjuvants in free suspension. This slower release can improve DC uptake and prolong DC activation state, with no notable cytotoxic effects [288]. Importantly, any imposed or induced change in the immune system raises concerns of toxicity and adverse effects. For this reason, developments in nano-immunotherapy should be complemented with appropriate toxicology studies to verify pharmacological safety. Fig. 3 presents a summary of nano-immunotherapy as described above.

*Investigative models for evaluating nano-immunotherapy* Suitable models of the TME are needed to develop and test immunotherapeutic NPs. Murine models are commonly used to evaluate NPs *in vivo*, and are a powerful system to determine NP organ uptake and distribution. Furthermore, mice can be used to observe the effects of NPs over several months, and this may not be possible with traditional *in vitro* systems. Mouse models offer an edge over two-dimensional studies, which can bring about disparities in cell response or drug sensitivity as they fail to recapitulate the complex three-dimensional architecture of the TME [289]. However, mouse models inevitably raise ethical issues, are costly and time-consuming, and also exhibit species-related differences from a human cancer setting.

Microfluidic models of the cancer TME could represent a vital intermediate step to bridge *in vitro* high throughput screenings, animal studies and clinical patient trials. The several advantages of microfluidic platforms for immune-cancer study have been extensively reviewed elsewhere [290]. Importantly, these platforms have been successfully applied to test the efficacy of different immunotherapies, including engineered T cells [291], ICB [292], gene silencing of checkpoint proteins [293], and metabolic reprograming of TAMs [294]. Through their rational design, microfluidic platforms can also be used to help understand and characterize NP transport and NP physicochemical properties [[295], [296], [297]].

To conclude, research efforts should continue to boost the efficacy of NP-based immunotherapies to justify their usefulness over existing therapeutic regimes. The development and testing of combined immunotherapies should also consider that a set of different NP designs, instead of a single design strategy, may be necessary to achieve optimal results for the patient. The future of nano-immunotherapy will need to consider and account for several dynamic interdependent relationships. These include the interdependence between the physicochemical parameters of NPs and the resultant protein corona, the interaction between the *in vivo* biological NP entity and specific immune-cell subsets as well as the patient-specific immune landscape and overall therapeutic efficacy over the course of treatment. As such, greater clinical impact may be reached through fostering collaborations between *in vitro* and/or *in vivo* studies (with more extensive characterizations of *in vivo* NP properties), and *in silico* predictions of the numerous interdependent relationships that inevitably govern the patient-specific success of nano-immunotherapy.

## 4 Concluding remarks

Advances in immunotherapy are leading the way in cancer treatment, with successes already apparent in many cancer types. The most accomplished immunotherapeutics are checkpoint inhibitors, which have been remarkable in treating cancers such as NSCLC, melanoma and pancreatic cancer. However, suppressing T cell activity through immune checkpoints is just one of the many immune-evasive mechanisms adopted by cancer. Reports of non-responders to checkpoint inhibitors [298] clearly suggest a need for alternative immunotherapeutic approaches. It is imperative to acknowledge that each tumor, even with similar underlying histology, may have evolved a unique strategy to evade immune control. Therefore, delineating the plethora of mechanisms of tumor evasion is essential to develop and administer the most effective cancer treatment. For example, ACT can be administered to patients with a low TIL or NK count. Combined therapies based on checkpoint inhibitors and ACT have yielded durable responses in patients diagnosed with refractory melanoma [130], metastatic melanoma [297] and breast cancer [299]. Alternatively, targeting immune metabolism can transform the TME to be pro-inflammatory and relieve immunosuppression. With the development of many immunotherapeutic options targeting various aspects of tumor immunity, more research efforts are essential to examine the possibility and impact of different combinations of immunotherapies on cancer outcomes.

## Competing financial interests

The authors have no competing financial interests to declare.
